# Impact of Intestinal Colonization With *Clostridioides difficile* on Clinical Outcomes in Alcohol-Associated Hepatitis

**DOI:** 10.14309/ctg.0000000000001084

**Published:** 2026-08-17

**Authors:** James Vu, Isaac Swartzman, Robert L. Pecha, Scott Crabtree, Jordan Holliday, Vikrant Rachakonda

**Affiliations:** 1 Department of Internal Medicine, University of California, Davis, Sacramento, California, USA;; 2 Division of Gastroenterology and Hepatology, University of California, Davis, Sacramento, California, USA;; 3 Division of Infectious Disease, University of California, Davis, Sacramento, California, USA.

**Keywords:** intestinal dysbiosis, bile acids, infection, ascites, hepatic encephalopathy, corticosteroids

## Abstract

**INTRODUCTION:**

*Clostridioides difficile* (*C. difficile*) is associated with reduced intestinal microbial diversity, and evidence links dysbiosis to increased disease severity in alcohol-associated hepatitis (AH). We therefore evaluated *C. difficile* colonization (CDC) as a predictor of clinical outcomes in AH.

**METHODS:**

We conducted a retrospective cohort study of 471 patients hospitalized with AH who underwent *C. difficile* surveillance from July 2017 to September 2024. CDC was defined as positive toxigenic *C. difficile* rectal swab PCR without symptoms. Patients were followed for 90 days after admission.

**RESULTS:**

Sixty-nine patients with AH (14.7%) had CDC. Notably, 163 (34.6%) developed infections, and infection rates were higher in CDC patients [53.6% vs 31.3%, *P* = 0.001], including *C. difficile* infection (CDI, 8.7% vs 0.8%, *P* < 0.01). Patients with CDC had more ascites (78.3% vs 22.5%, *P* < 0.001), gastrointestinal (GI) bleeding (39.1% vs 27.9%, *P* = 0.042), and ICU admission (46.4% vs 34.6%, *P* = 0.042), but MELD 3.0 was similar between groups (28 [IQR 23–35] vs 26 [IQR 21–31], *P* = 0.065). Ninety-day survival was lower with CDC (66.7 [95% CI 58.9–74.4] days vs 77.2 [95% CI 74.6–79.9] days, *P* = 0.007). Age (aHR 1.032, 95% CI: 1.012–1.052; *P* < 0.001), MELD 3.0 (aHR 1.083, 95% CI: 1.056–1.111; *P* < 0.001) and CDC (aHR 1.369, 95% CI: 1.035–2.245; *P* = 0.041) were associated with 90-day mortality.

**DISCUSSION:**

In AH, CDC is associated with more infection, ascites, gastrointestinal bleeding, and ICU admission, and independently predicts reduced 90-day survival. CDC may serve as a novel marker of disease severity and infection risk in AH.

## Graphical abstract

**Figure:**
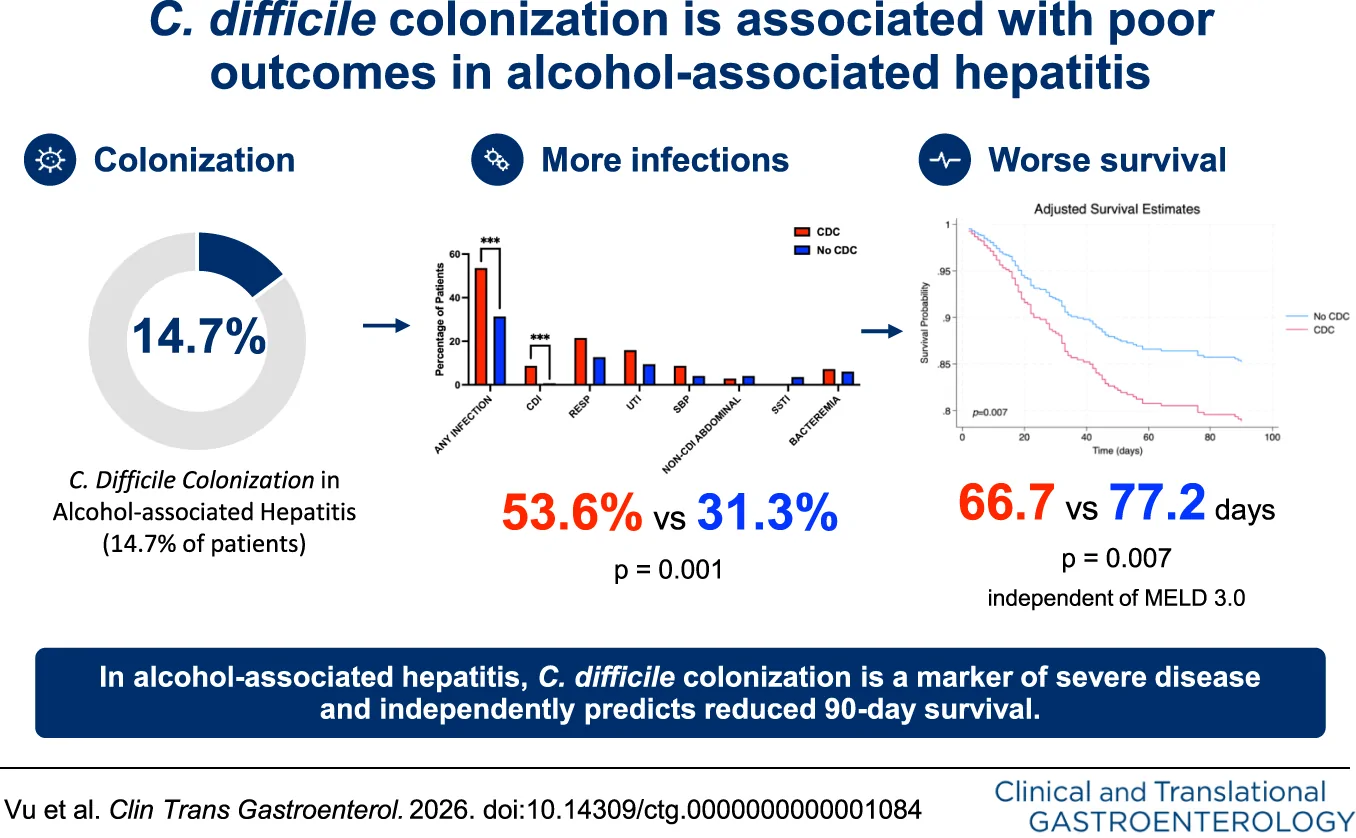

## INTRODUCTION

Alcohol-associated liver disease (ALD) is a form of steatotic liver disease that develops in the setting of sustained alcohol use. Alcohol-associated hepatitis (AH) is a severe form of ALD characterized by abrupt onset of jaundice with high rates of infections, intrahepatic and extrahepatic organ failures; AH is a highly lethal condition, with a short-term mortality of approximately 30%–50% (1–4).

The pathophysiology of AH is complex and incompletely understood. In the liver, enzymes such as alcohol dehydrogenase and CYP2E1 facilitate ethanol metabolism into acetaldehyde and other reactive oxygen species. These metabolites can cause hepatocyte injury, leading to innate immune activation by damage-associated molecular patterns (DAMPs) that trigger an inflammatory cascade (1,5,6). Outside of the liver, alcohol has been found to increase intestinal permeability, alter bile acid (BA) enterohepatic circulation, promote dysbiosis (increases in *Proteobacteria* and *Fusobacteria*, reductions in *Bacteroidetes* and *Lactobacillus*, increases in candidal species and decreases in overall fungal diversity) and drive translocation of gut bacterial peptides such as lipopolysaccharide and pathogen-associated molecular patterns (PAMPs). This process contributes to worsening hepatic inflammation and changes in metabolic profiles (7–9).

*Clostridioides difficile *colonization (CDC) is common and is seen in up to 21% of hospitalized patients in the Unites States. (10,11). CDC does not occur alone, but is associated with many other alterations in the microbiome that could potentially be deleterious in ALD (12–14). The transition from CDC to *C. difficile* infection (CDI) occurs when *C. difficile *proliferates and produces toxins A, B, and binary toxin. Both CDC and CDI are commonly facilitated by antibiotics and subsequent dysbiosis but can also occur in the setting of decreased secondary BAs (with subsequently increased taurocholic acid), proton pump inhibitor (PPI) use, and corticosteroid use (11,15,16). Although the incidence of CDI has overall decreased, recurrent CDI has increased significantly in the past few years (11).

In 2017, our institution implemented hospital-wide screening for colonization with toxigenic *C. difficile* in all hospitalized patients to reduce rates of transmission and infection. CDI has been clearly associated with poor outcomes in patients with AH (14); however, the implications of CDC without infection among patients with AH have not been fully assessed (17–19). Our study aimed to evaluate the impact of CDC on outcomes among patients hospitalized with AH.

## METHODS

### Study design and population

We conducted a retrospective cohort study of patients admitted with acute AH who underwent *C. difficile *surveillance at University of California Davis Medical Center (UCDMC) between July 2017 and September 2024. Patients 18–80 years of age were eligible for inclusion if they met diagnostic criteria for probable or definite AH as defined by National Institute on Alcohol Abuse and Alcoholism which include: (i) onset of jaundice within the previous 8 weeks, (ii) ongoing alcohol consumption, (iii) serum bilirubin >3 mg/dL, and (iv) aspartate aminotransferase (AST) to alanine aminotransferase (ALT) ratio >1.5 with both enzymes <400 IU/L or (v) liver biopsy confirming diagnosis of AH. Exclusion criteria included abstinence from alcohol for > 6 weeks before admission, histologic or serologic evidence of another liver disease besides AH, and previous solid organ or hematopoietic transplant. Although AH has a heterogeneous presentation, patients were typically hospitalized for severe disease warranting consideration of corticosteroid therapy and inpatient monitoring, hepatic decompensation (worsening jaundice, hepatic encephalopathy, ascites, or gastrointestinal bleeding), acute kidney injury (AKI), suspected or confirmed infection, or a need for supportive care such as management of alcohol withdrawal; because the study was retrospective, a single documented admission indication was not captured for every patient.

All research was conducted in accordance with both the Declarations of Helsinki and Istanbul. This study was approved by the institutional review board at UCDMC Model for End Stage Liver Disease.

### *C. difficile* surveillance and definitions

As part of a systematic hospital screening protocol, patients at UCDMC undergo rectal swab testing at admission for toxigenic *C. difficile* tcdB DNA polymerase chain reaction (PCR) (Xpert *C. difficile* test; Cepheid). CDC was defined as a positive rectal swab PCR for *C. difficile* in the absence of symptoms consistent with CDI. The diagnostic criteria for CDC used in our study are similar to definitions of CDC from other published studies (10,20). CDI was defined as 3 or more unformed stools in a 24 hour period in the setting of positive *C. difficile* tcdB DNA PCR and/or stool toxin EIA.

### Clinical and demographic evaluation

Age, biological sex, ethnicity, and primary language were recorded, and patient survival up to 90 days after hospitalization were determined from clinical chart review and the Social Security Death Index. Laboratory data were obtained on the first day of hospital admission and included serum albumin, total bilirubin, creatinine, sodium, international normalized ratio, platelet count, white blood cell count (WBC) with differential, neutrophil-to-lymphocyte ratio. Model for End-Stage Liver Diseases (MELD) and MELD 3.0 were calculated as previously described (21,22). Clinical complications of AH including AKI, gastrointestinal (GI) bleeding, ascites, and hepatic encephalopathy, were recorded. Usage rates of corticosteroids, PPI, lactulose, and antibiotics including rifaximin, were recorded. Corticosteroid use reflects inpatient administration for AH; systemic corticosteroid exposure before admission was not captured. Patients were then stratified by *C. difficile* colonization status. The primary outcome was 90-day survival. Secondary outcomes included development of infections (both non-CDI and CDI), 30-day readmission rates, and steroid utilization.

### Definitions of infections

The following diagnostic criteria were used to define infections. Urinary tract infection: abnormal urine sediment with >10 leukocytes/field, positive leukocyte esterase, and/or positive cultures in patients with (i) urinary symptoms including dysuria, urgency, frequency, or costovertebral or suprapubic discomfort (ii) symptoms/signs of systemic infection (fevers, chills, rigors, altered mental status, malaise); respiratory infections, including (i) *pneumonia*: chest imaging with evidence of pulmonary infiltrates in a patient with symptoms/signs of respiratory distress, cough, or systemic infection; (ii) *bronchitis*: clinical features of infection, cough and/or respiratory distress without infiltrates on chest imaging with positive sputum culture or viral nasal swab detection of influenza, coronavirus disease 2019, or other respiratory viruses; spontaneous bacterial peritonitis: ascitic fluid polymorphonuclear cell count > 250/mm^3^; skin and soft tissue infections: symptoms/signs of infection with associated swelling, tenderness, and erythema of the skin or imaging evidence of soft tissue abscess; non-CDI abdominal infections, including (i) Cholangitis: cholestasis and/or jaundice, signs/symptoms of systemic infection, and/or right upper quadrant pain with radiologic evidence of biliary obstruction; (ii) Colitis: abdominal pain with change in bowel habit and imaging evidence of either ileus, colonic dilation, and/or colonic wall thickening with stranding, elevated fecal calprotectin level and/or positive stool multiplex nucleic acid detection of bacterial infections (GI BioFire, bioMeriux, Plainview, New York) or positive stool culture for agents other than *C. difficile*; (iii) Secondary bacterial peritonitis: ascites polymorphonuclear cell count > 250/mm^3^ and/or positive ascitic fluid culture in a patient with imaging evidence of an intestinal or biliary source of infection; bacteremia: positive blood cultures for pathogenic organisms.

### Statistical analysis

Statistical analysis was performed with Stata version 19 (StataCorp, College Station, TX) and GraphPad Prism Version 10 (GraphPad, San Diego, CA). Clinical and demographic variables were reported as medians with interquartile ranges (IQRs, 25th–75th quartiles), absolute frequencies, and percentages. For continuous variables, comparisons between groups were performed using nonparametric Mann-Whitney *U* or Kruskal Wallis tests with post hoc Dunn test for multiple comparisons. Fisher exact tests were used to compare categorical variables between groups. For analysis of primary outcomes including 90-day transplant free survival, patients were right-censored at 90 days. Time to event data were analyzed using Kaplan-Meier analysis, and survival curves compared between groups using log-rank tests. Prognostic factors of overall survival were analyzed with Cox proportional hazards models. To assess predictors of infections, logistic regression models were performed. For both Cox analysis and logistic regression, covariates on univariate analysis with *P* < 0.05 were then included in multivariate models. A *P*-value < 0.05 was considered statistically significant for all analyses.

Because this was a retrospective study, a post hoc justification of sample size was performed. Among the 69 patients identified with CDC, crude 90-day mortality was 34.8%. Using a 2-sided, 2-sample z-test for independent proportions with unequal group sizes, following the approach of Chow et al (23), the CDC group size (n1) was fixed at 69, with expected proportions of p1 = 0.348 and p2 = 0.211, a significance level of α = 0.05, and power of 80%. Solving the standard 2-proportion sample-size formula for the required allocation ratio yielded a minimum of 209 uncolonized patients; with 402 uncolonized subjects available, the cohort was adequately powered to detect a difference in this outcome.

## RESULTS

### Baseline characteristics

Baseline clinical and demographic characteristics are summarized in Table 1. A total of 582 patients were hospitalized for AH during the study period. Of these, 471 patients (80.9%) successfully completed CDC surveillance stool testing and met inclusion criteria. Among tested patients, 69 (14.7%) had CDC, and 402 (85.3%) did not; from 2017 to 2024, the overall rate of CDC was 6.4% at our hospital. There were no differences in age, sex, race, primary language, hospital length of stay, or 30-day readmission rates between cohorts. Although corticosteroid use and PPI use did not differ between colonized and uncolonized patients, the CDC group had higher rates of rifaximin use (47.8% vs 35.3%, *P* = 0.033), and lactulose use (78.3% vs 64.7%, *P* = 0.017). Overall, 346 patients (73.5%) received antibiotics, and antibiotic use was higher in patients with CDC (85.5% vs 71.4%, *P* = 0.008). Among patients receiving antibiotics, 164 (59.4%) had no infection diagnosed, and there were no differences between CDC and uncolonized groups.

**Table 1.** Baseline demographic and clinical characteristics stratified by *C.difficile* colonization status

Feature	All (N = 471)	CDC (N = 69)	NO CDC (N = 402)	*P* value
Age, y (median, IQR)	45 (37–54)	47 (40–54)	44 (37–54)	0.1403
Female (n,%)	197 (41.8%)	28 (40.6%)	169 (42.0%)	0.464
White (n, %)	214 (45.4%)	35 (50.7%)	179 (44.2%)	0.205
English (n, %)	431 (91.5%)	60 (86.9%)	371 (92.8%)	0.112
LOS, d (median, IQR)	7 (4–12)	8 (5–13)	7 (4–11)	0.1035
ICU (n, %)	171 (36.3%)	32 (46.4%)	139 (34.6%)	0.042
Steroids (n, %)	91 (19.3%)	13 (18.8%)	78 (19.4%)	0.532
30-d readmission, n (%)	87 (18.56%)	15 (21.7%)	72 (17.9%)	0.273
PPI (n, %)	359 (76.2%)	55 (79.7%)	304 (75.6%)	0.284
Antibiotics (n, %)	346 (73.5%)	59 (85.5%)	287 (71.4%)	0.008
Lactulose (n, %)	314 (75.2%)	54 (78.3%)	260 (64.7%)	0.017
Rifaximin (n, %)	175 (37.2%)	33 (47.8%)	142 (35.3%)	0.033

Continuous variables are presented as medians with interquartile range (IQR).

CDC, *C. difficile* colonization; ICU, intensive care unit; LOS, length of stay; PPI, proton pump inhibitor.

### CDC is associated with portal hypertensive complications in AH

Although MELD and MELD 3.0 trended higher in the CDC group, this difference did not reach statistical significance (MELD: 23 [20–31] vs 22 [18–27], *P* = 0.062; MELD 3.0: 28 [23–35] vs 26 [21–31], *P* = 0.055, Table 2), and there was no difference in the proportions of patients with severe AH (defined by MELD > 20) between groups (66.7% vs 59.5%, *P* = 0.159). There were no significant differences in serum bilirubin, international normalized ratio, creatinine, or sodium between groups, but CDC patients had lower serum albumin levels (2.4 [2.0–3.0] g/dL vs 2.8 [2.3–3.3] g/dL, *P* = 0.001, Table 2). Ascites (78.2% vs 57.0%, *P* < 0.001) and GI bleeding (39.1% vs 27.9%, *P* = 0.042) occurred more frequently in CDC patients, whereas rates of AKI and hepatic encephalopathy were similar between groups (Figure 1a). Finally, ICU admission rates were higher in the CDC cohort (46.4% vs 34.6%, *P* = 0.042, Table 1). Together, these findings suggest that despite similar degrees of hepatic dysfunction between groups, CDC is associated with more pronounced portal hypertensive complications.

**Table 2.** Baseline clinical laboratory markers of disease severity stratified by *C. difficile* colonization

Feature	All (N = 471)	CDC (N = 69)	NO CDC (N = 402)	*P* value
MELD (a.u.)	22 (18–28)	23 (20–31)	22 (18–27)	0.062
MELD 3.0 (a.u.)	26 (21–32)	28 (23–35)	26 (21–31)	0.055
Bilirubin	8.0 (5.3–17.4)	8.6 (5.3–17.4)	7.8 (4.9–15.3)	0.214
INR	1.82 (1.50–2.25)	1.87 (1.58–2.37)	1.81 (1.49–2.21)	0.187
Creatinine	0.89 (0.66–1.43)	0.99 (0.67–1.89)	0.88 (0.66–1.38)	0.124
Sodium	133 (129–136)	133 (128–137)	133 (129–136)	0.874
Albumin	2.7 (2.2–3.3)	2.4 (2.0–3.0)	2.8 (2.3–3.3)	0.001
WBC (10^9^/L)	8.6 (6.0–13.3%)	11.0 (6.8–14.8)	8.4 (5.9–13.1)	0.036
NLR (a.u.)	7.2 (3.9–12.8)	8.2 (4.6–14.8)	7.2 (3.9–12.3)	0.281

Continuous variables are presented as medians with IQR.

INR, international normalized ratio; IQR, interquartile range; MELD, model for end stage liver diseases; NLR, neutrophil/lymphocyte ratio, WBC, white blood cell count.

**Figure 1.**
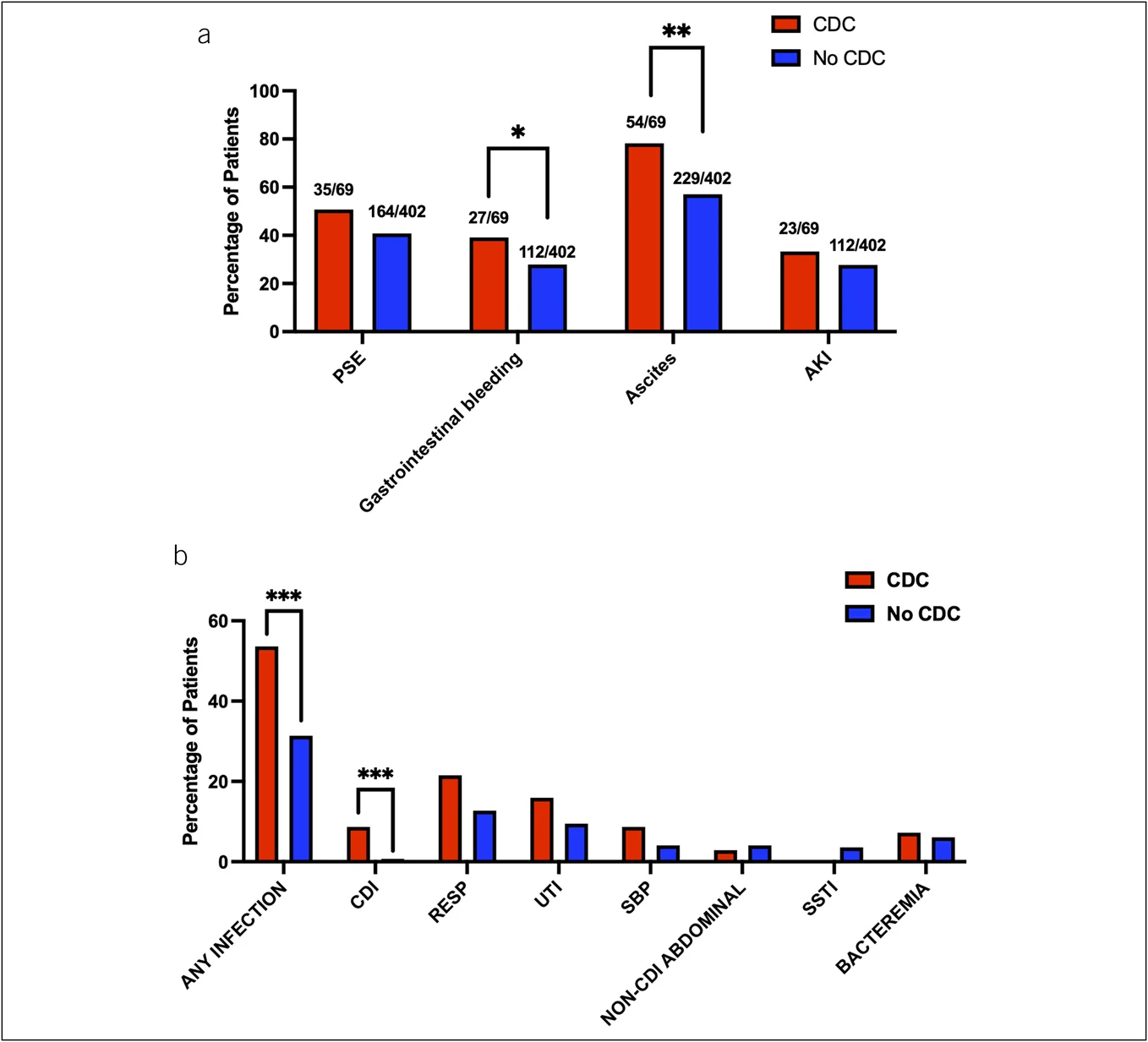
Complications of alcohol-associated hepatitis stratified by *Clostridioides difficile* colonization status, including (**a**) portal hypertensive complications and (**b**) infections. *P < 0.05, **P < 0.01, ***P < 0.001. AKI, acute kidney injury; CDC, *C. difficile* colonization; CDI, *C. difficile* infection; PSE, portosystemic encephalopathy; SBP, spontaneous bacterial peritonitis; SSTI, skin or soft tissue infection.

### CDC is associated with higher rates of infection in patients with AH

Clinical features of infections are summarized in Figure 1b and Supplementary Table 1 (see Supplementary Digital Content, https://links.lww.com/CTG/B584). Overall, 163 patients (34.6%) developed infections, and infection rates were higher in CDC patients (53.6% vs 31.3%, *P* = 0.001). The most frequently encountered infections were respiratory infections (13.8% of patients), followed by urinary tract infection (10.4%), spontaneous bacterial peritonitis (4.7%), bacteremia (6.2%), non-CDI intestinal infections (3.8%), and skin and soft tissue infections (3.0%). Nine patients (2.2%) developed CDI, and as demonstrated in other studies (17), CDC was associated with higher rates of progression to symptomatic CDI (8.7% vs 0.8%, *P* < 0.001, Figure 1b). A bacterial organism was cultured in 77/163 (47.2%) of patients with infections, and among these, 28 (36.3%) were drug-resistant organisms. Rates of drug-resistant organisms, however, did not differ between groups (19/37 [51.4%] vs 58/126 [46.0%], *P* = 0.351). There were no significant differences in infection rates between steroid treated and steroid naïve patients (26/91 [28.6%] vs 137/380 [36.1%], *P* = 0.109), but among steroid-treated patients, infections were more common in the CDC group (7/13 [53.8%] vs 19/78 [24.4%], *P* = 0.036).

We then used logistic regression to assess factors associated with infection development in our study population. On univariate analysis, female sex, ICU admission, ascites, encephalopathy, GI bleeding, AKI, MELD 3.0, WBC, PPI use, rifaximin use, lactulose use and CDC were associated with development of infection (Table 3). However, in multivariate models, only female sex (OR 1.649 [95% white blood cell count [CI] 1.065–2.554], *P* = 0.025), ICU admission (OR 2.852 [95% CI 1.619–4.117], *P* < 0.001), AKI (odds ratio [OR] 1.766 [95% CI 1.063–2.936], *P* = 0.028), and CDC (OR 2.533 [95% CI 1.198–3.806], *P* = 0.010) were independently associated with infection (Table 3). Together these findings suggest that CDC is an important contributor to infection risk in AH.

**Table 3.** Univariate and multivariate logistic regression analyses for factors associated with infection among patients with successfully completed toxigenic *C. difficile* colonization testing (n = 402)

Feature (for infections only)	UV OR	*P* value	MV OR	*P* value
Age	1.013 (0.995–1.030)	0.152		
Sex	1.514 (1.032–2.222)	0.034	1.649 (1.065–2.554)	0.025
White	0.825 (0.563–1.210)	0.325		
ICU	3.602 (2.413–5.377)	<0.001	2.582 (1.619–4.117)	<0.001
Platelets	0.999 (0.998–1.002)	0.749		
Ascites	2.292 (1.524–3.447)	<0.001	1.166 (0.710–1.917)	0.543
Hepatic encephalopathy	1.855 (1.262–2.724)	0.002	1.014 (0.642–1.600)	0.953
Variceal bleed	1.689 (1.122–2.540)	0.012	0.977 (0.599–1.595)	0.926
AKI	2.814 (1.861–4.255)	<0.001	1.766 (1.063–2.936)	0.028
MELD 3.0	1.063 (1.040–1.088)	<0.001	1.005 (0.976–1.036)	0.725
Steroids	0.709 (0.430–1.171)	0.179		
WBC	1.052 (1.024–1.081)	<0.001	1.028 (0.997–1.059)	0.074
NLR	1.020 (0.999–1.041)	0.058		
CDC	2.533 (1.509–4.251)	<0.001	2.136 (1.198–3.806)	0.010
PPI	2.317 (1.407–3.815)	0.001	1.409 (0.792–2.507)	0.243
Rifaximin	2.748 (1.853–4.076)	<0.001	1.527 (0.913–2.556)	0.107
Lactulose	2.655 (1.702–4.142)	<0.001	1.460 (0.812–2.627)	0.206

AKI, acute kidney injury; CDC, *C. difficile* colonization; ICU, intensive care unit; LOS, length of stay; NLR, neutrophil-lymphocyte ratio; OR, odds ratio; PPI, proton pump inhibitor; WBC, white blood cell count.

### CDC is associated with reduced 90-day survival in AH

Patients were followed for up to 90 days after admission, and subjects still living without transplantation were censored at 90 days. Over the study period, there were 109 events (23.5% of patients), including 106 deaths and 3 liver transplants.

We then used Cox proportional hazards regression to determine factors associated with increased mortality in the study population (Table 4). On univariate analysis, age, ICU admission, ascites, hepatic encephalopathy, GI bleed, AKI, MELD 3.0, antibiotic administration, infection, WBC, neutrophil-to-lymphocyte ratio and CDC were associated with increased 90-day mortality. On multivariate analysis, however, only age, MELD 3.0, and CDC remained associated with increased 90-day mortality. CDC independently predicted 90-day mortality (adjusted hazards ratio [aHR] 1.369, 95% CI: 1.035–2.245; *P* = 0.041), even after adjusting for age (adjusted HR 1.032, 95% CI: 1.012–1.052; *P* < 0.001) and disease severity using MELD 3.0 (aHR 1.083, 95% CI: 1.056–1.111; *P* < 0.001, Table 4). Raw mortality rates were significantly higher in the CDC group (34.8% vs 21.1%, *P* = 0.010. Figure 2a). In Kaplan-Meier analyses, CDC was associated with lower 90-day survival (66.7 days [95% Cl: 58.9–74.4] vs 77.2 days [95% Cl: 74.6–79.9], *P* = 0.007, Figure 2b).

**Table 4.** Univariate and multivariate Cox proportional hazards models for factors associated with 90-day survival among patients with successfully completed toxigenic *C. difficle* colonization testing (n = 471)

Feature (90 d mortality)	UV HR	*P* value	MV HR	*P* value
Age	1.037 (1.020–1.055)	<0.001	1.032 (1.012–1.052)	0.001
Sex	0.794 (0.537–1.173)	0.242		
White	1.005 (0.688–1.468)	0.978		
ICU	3.077 (2.095–4.518)	<0.001	1.354 (0.847–2.164)	0.205
Platelets	0.999 (0.997–1.001)	0.557		
Ascites	3.947 (2.379–6.549)	<0.001	1.681 (0.933–3.029)	0.084
Hepatic encephalopathy	2.177 (1.484–3.192)	<0.001	1.149 (0.750–1.762)	0.523
Variceal bleed	2.377 (1.627–3.469)	<0.001	1.227 (0.789–1.909)	0.363
AKI	3.111 (2.132–4.539)	<0.001	1.093 (0.698–1.712)	0.698
MELD 3.0	1.099 (1.079–1.119)	<0.001	1.083 (1.056–1.111)	<0.001
Antibiotics	4.079 (2.128–7.820)	<0.001	1.315 (0.590–2.932)	0.504
Infection	2.716 (1.854–3.963)	<0.001	1.456 (0.936–2.266)	0.095
WBC	1.023 (1.004–1.042)	0.017	0.999 (0.972–1.027)	0.948
NLR	1.020 (1.011–1.030)	<0.001	0.999 (0.984–1.014)	0.887
CDC	1.840 (1.168–2.896)	0.008	1.369 (1.035–2.245)	0.041

AKI, acute kidney injury; CDC, *C. difficile* colonization; HR, hazards ratio; ICU, intensive care unit; LOS, length of stay; NLR, neutrophil-lymphocyte ratio; PPI, proton pump inhibitor; WBC, white blood cell count.

**Figure 2.**
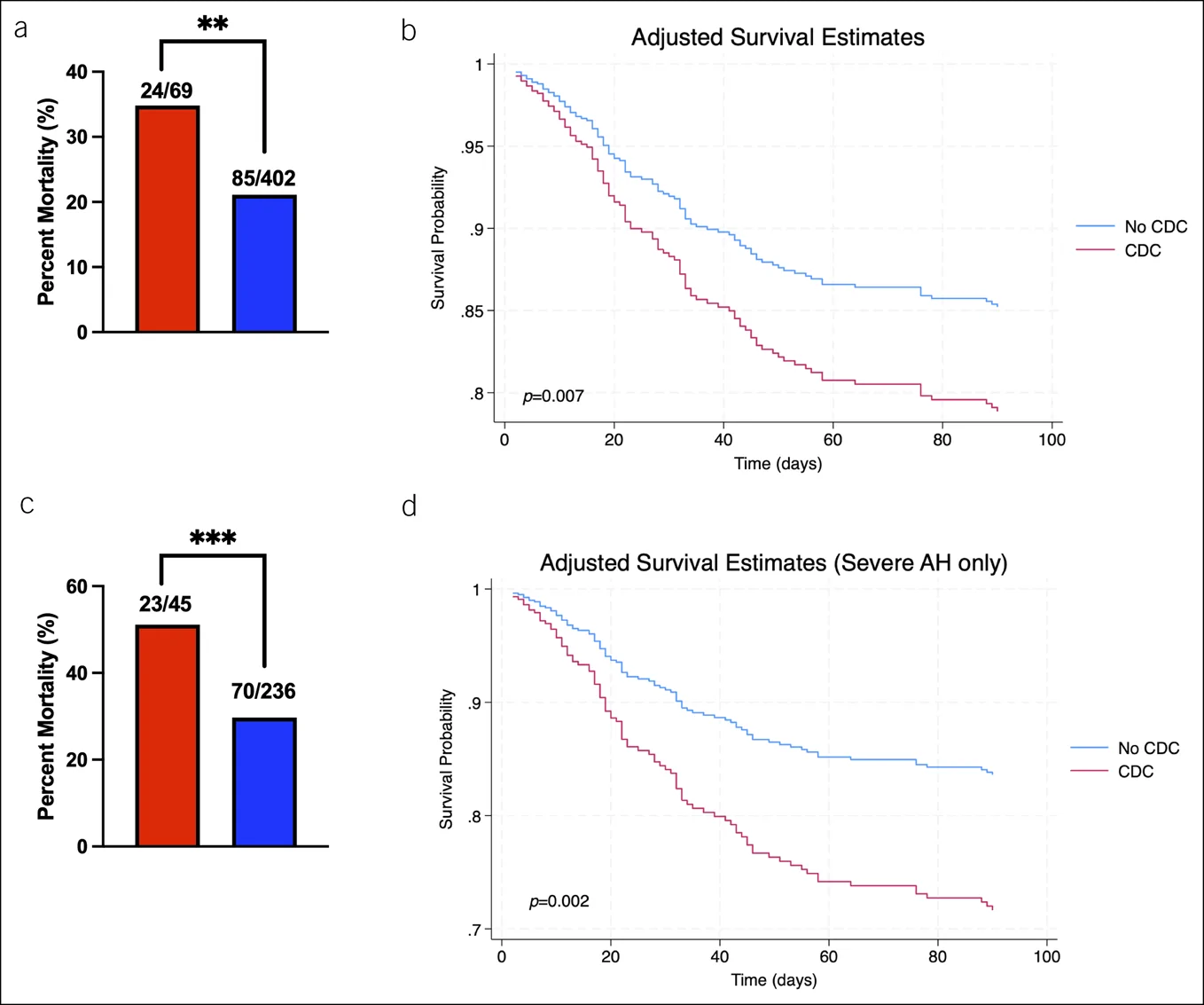
Association between *Clostridioides difficile* colonization status and 90-day mortality in (i) all patients in (**a**) raw proportions and (**b**) Cox proportional hazards curves adjusted for other significant covariates (age, ascites, MELD 3.0, and infections) and (**ii**) patients with severe alcohol-associated hepatitis (defined as MELD > 20) in (**c**) raw proportions and (**d**) Cox proportional hazards curves adjusted for other significant covariates (age, ICU admission, and model for end stage liver diseases 3.0). **P < 0.01; ***P < 0.001. AH, alcohol-associated hepatitis; CDC, *C. difficile* colonization.

To assess the influence of CDC on survival in critically ill patients with AH, we performed an additional subgroup analysis in patients with severe AH, defined as MELD > 20. In this cohort, only age (adjusted HR 1.029 [95% CI 1.010–1.049], *P* = 0.002), ICU admission (adjusted HR 1.589 [95% CI 1.027–2.460], *P* = 0.037), MELD 3.0 (adjusted HR 1.086 [95% CI 1.060–1.114], *P* < 0.001), and CDC (adjusted HR 1.866 [95% 1.153–3.005], *P =* 0.010) were independently associated with increased 90-day mortality (see Supplementary Table 2, Supplementary Digital Content, https://links.lww.com/CTG/B584 and Figure 2c,d). Among subjects with severe AH, those with CDC had shorter 90-day survival compared with uncolonized patients (55.5 days [95% CI 45.2–65.7] vs 72.4 days [95% CI 68.6–76.2], *P* = 0.002). Together these findings suggest that colonization with *C. difficile* is associated with 90-day mortality in AH independent of liver disease severity and other portal hypertensive complications.

## DISCUSSION

In this retrospective study, we assessed the impact of CDC on clinical outcomes in patients hospitalized with AH. Four important findings were noted. First, CDC rates in AH were more than 2 times greater than overall hospital colonization rates. Second, patients with CDC had higher rates of ICU admission and more advanced portal hypertensive complications, including ascites and GI bleeding. Next, patients with CDC experienced more infections than uncolonized subjects, and CDC was independently associated with infection risk in AH. Finally, CDC was independently associated with 90-day mortality, and this risk was enhanced among patients with severe AH.

The prevalence of CDC among patients with AH was 14.7%, whereas the overall prevalence of CDC in all hospitalized patients at UCDMC over the study period was only 6.4%. Although published studies on CDC prevalence in AH are limited, our findings are similar to reports from a large analysis of the national inpatient sample, which demonstrated significantly higher rates of CDI in AH compared with other hospitalized patients (17). Furthermore, Yan et al reported that toxigenic CDC was found in 19.8% of patients hospitalized for decompensated cirrhosis, with significantly higher rates of colonization seen in patients with more advanced liver disease (17,24). Patients with AH often exhibit well-established risk factors of both CDC and CDI including repeated healthcare encounters before index hospitalization (20), increased antimicrobial usage, and PPI therapy (12,21). In this study, we identified higher rates of antimicrobial use in CDC patients. Overall, antibiotics and PPI were used in 73.2% and 76.2% of all patients, and this may also contribute to higher rates of CDC in hospitalized patients with AH.

The medication associations observed in our cohort merit comment. Although PPI use is frequently cited as a risk factor of *C. difficile*, it did not differ by colonization status, whereas rifaximin and lactulose use were more common among colonized patients. Several factors likely account for this pattern. The reported association between PPI and *C. difficile* is predominantly from studies of symptomatic infection (25), and recent work suggests this relationship is substantially attenuated after accounting for concurrent antibiotic exposure (26). Moreover, PPIs were used in 76.2% of the cohort, limiting the contrast available to detect an independent effect. Regarding rifaximin and lactulose, their usage may serve as markers of more advanced disease with recurrent hepatic encephalopathy, and reported changes in the gut microbiome of patients with hepatic encephalopathy are similar to those observed in CDC and CDI (27–29). Finally, studies suggest that lactulose suppresses *C. difficile* growth as a prebiotic (30), and rifaximin exposure has been associated with reduced CDI risk in cirrhosis (31), although resistance and breakthrough infection have also been described (32). These observations argue against a direct medication effect and favor confounding by underlying disease severity.

AH is characterized by an intestinal microbial environment conducive to both CDC and CDI. Compared with heavy drinking controls, patients with AH exhibit decreased alpha and beta diversity with reductions in butyrate-producing bacterial genera, including *Lachnospiraceae* and *Ruminococcaceae* (33,34), and similar intestinal microbial alterations have been observed in both CDC and CDI (13,29,35). Our group and others have reported increased levels of serum and fecal primary BAs with decreased secondary BAs in AH (8,36); these alterations in the intestinal BA pool may promote CDC, because primary BAs promote, whereas secondary BAs inhibit, germination of *C. difficile* spores (37). These alterations in the intestinal microbial and nutrient environment likely lead to predisposition to CDC in patients with AH.

In our cohort, patients with CDC experienced more sequelae of portal hypertension, including ascites and GI bleeding. This result aligns with a growing body of evidence that gut-liver axis dysfunction can have clinical significance in advanced liver disease by increasing portal pressures (38,39). This relationship is likely bidirectional because (i) portal hypertension leads to mechanical disruption of intestinal tight junctions and increased intestinal permeability, and (ii) intestinal dysbiosis leads to loss of healthy commensal bacteria that support the intestinal mucus layer, whereas a simultaneous increase in pathogenic organisms leads to intestinal translocation of bacteria and PAMPs. These PAMPs then activate the intrahepatic macrophage inflammasome by Toll-like receptor signaling, leading to intrahepatic inflammation, fibrosis, and increased sinusoidal vascular resistance (40–42). Whether CDC contributes directly to, or merely reflects pathogenic intestinal dysbiosis, however, remains unclear, and further studies would need to evaluate the influence of CDC on portal pressures (7–9,14,43). Notably, the rates of hepatic encephalopathy did not differ between the groups. This raises questions about the complex interaction between the gut and liver regarding clearance of ammonia, along with questions of whether *C. difficile* alters intestinal ammonia handling. It has been shown in previous studies that *C. difficile* can modulate pH by ammonium ion epithelial cell transporters by toxin B, resulting in a more alkaline pH in patients with CDI (44,45). Lactulose on the other hand has the opposite effect on stool, decreasing the pH. In our study, CDC patients had similar rates of encephalopathy despite signs of worse portal hypertension when compared with noncolonized patients, suggesting that other mechanisms besides intestinal pH drive interactions between CDC, lactulose use, and hepatic encephalopathy.

In our AH population, CDC was associated with a higher prevalence of infections compared with noncolonized patients. Although the precise etiology of this is unclear, there is significant overlap between risk factors of CDC and other nosocomial infections including healthcare exposure, immunocompromised condition, corticosteroid use, and antibiotic use (10,46–48). Although less established, this may also reflect the significance of CDC as a marker of a dysregulated intestinal microbiome, which has been shown to play a role in systemic immune responses to nonintestinal infections (49). In addition, alcohol use is a known risk factor of pneumonia (50), and is also known to disrupt intestinal integrity and promote dysbiosis, which may contribute to the translocation of microbial products and systemic immune activation in the liver (5–9,50). Studies have demonstrated that there is similarity in the intestinal microbial profiles between colonized individuals and those with overt CDI–including decreased microbial diversity and increased *Proteobacteria*–a key marker of dysbiosis (12,13). It is possible that CDC may exacerbate these effects by further altering microbiome diversity and thus increase intestinal permeability, even in the absence of symptomatic CDI (7,10,12–14).

The relationship between *C. difficile* colonization and colonization with other multidrug-resistant organisms (MDRO) also warrants consideration. At our institution, screening for MDRO, including carbapenem-resistant *enterobacterales*, is performed for high-risk groups, including those previously residing in nursing or long-term acute care facilities, with previous MDRO or carbapenem-resistant *enterobacterales* infection, and patients admitted electively for liver transplantation. Two recent prospective cohort studies, in which ALD and AH were leading etiologies of liver disease, demonstrated an association between MDRO colonization and mortality (51,52). The relationship between CDC and MDRO colonization is less well defined, but recent work reported intestinal MDRO colonization in the stool of 20.8% of patients with CDI, and a separate study found that the odds of CDI were 3.38 times higher among patients with MDRO colonization than among uncolonized patients (53,54). Together, these findings suggest a bidirectional interaction between *C. difficile* and MDRO colonization, likely mediated by shared host risk factors such as immune dysfunction and previous antibiotic exposure.

Importantly, the presence of CDC was independently associated with reduced 90-day transplant-free survival, with Kaplan-Meier analysis showing a 10-day difference in mean survival. This result remained statistically significant after multivariate adjustment for age and MELD 3.0. In addition, CDC patients experienced significantly higher rates of ICU admission, further supporting the evidence that colonized patients tend to have more severe clinical trajectories. As noted earlier, previous findings suggest that CDI is associated with poorer outcomes in patients with AH or cirrhosis, and our findings suggest that even asymptomatic CDC may carry adverse prognostic significance (17–19).

Our results have important clinical implications. Although CDC is often regarded as a nonpathogenic state, our findings challenge that assumption in the context of AH and liver disease. The high prevalence and association with portal hypertensive complications, non-CDI infections, and mortality suggests that it may prove useful as an additional marker for risk stratification. In addition, our findings raise the question of whether targeted *C. difficile* prophylaxis in patients with AH with CDC would improve outcomes. A 2023 study by Louvet et al randomized 284 patients with AH to amoxicillin-clavulanate plus prednisolone or prednisolone alone, and found no significant difference in 60-day mortality; however, infections were significantly reduced in the antibiotic-treated cohort (55). Related to this finding, we found that among patients treated with corticosteroids for AH, CDC was associated with significantly increased rates of infection compared with uncolonized patients. Other studies have evaluated prophylactic rifaximin in AH, as well as norfloxacin in decompensated cirrhosis and acute-on-chronic liver failure, and found similar reductions in rates of infection without a consistent improvement in survival (56–58). Any potential benefit of antibiotic prophylaxis in this setting must be weighed against the risk that broad antimicrobial exposure itself promotes intestinal dysbiosis and predisposes to *C. difficile* colonization and infection, as reflected by the higher rates of antibiotic use among colonized patients in this study (8,12,13). However, no trial of patients with AH has been completed to date using oral vancomycin or fidaxomicin, and fecal microbiome transplant research continues with mixed results (59,60).

Our study has key strengths. We used both *ICD-10* codes and validated National Institute on Alcohol Abuse and Alcoholism criteria to accurately define the presence of AH, which increased the specificity of our cohort selection. In addition, CDC status was obtained through standardized PCR testing for toxigenic C. difficile, a reliable and objective measure of assessing colonization for toxigenic strains of C. difficile that confer increased risk of progression to CDI (61). Our measured clinical outcomes, including rates of infection, portal hypertensive complications and transplant-free survival, were both comprehensive and clinically relevant. Furthermore, the use of multivariate Cox proportional hazards modeling allowed for adjustment of potential confounders, thus strengthening the validity of our observed findings.

However, our study has limitations which must be acknowledged. First, this was a retrospective, single-center study which limits generalizability. Although CDC was associated with adverse clinical outcomes, this does not establish that colonization directly contributes to liver disease progression. Other confounding variables including number of previous healthcare visits, hospitalizations at other hospitals, and quantification of alcohol use before admission were not included in this study. In addition, our surveillance assay detects only toxigenic *C. difficile* and does not identify nontoxigenic strains, as nontoxigenic *C. difficile* is not a recognized risk factor of progression to CDI and may even be protective (43). However, nontoxigenic colonization may also contribute to intestinal dysbiosis, and future studies are planned to assess this contribution to AH pathophysiology.

Despite these limitations, our study is among the first to investigate CDC as a predictor of poor clinical outcomes and mortality in patients with acute AH. Our findings demonstrate that within this population, CDC was independently associated with significantly worse clinical outcomes, including higher rates of infection, ascites, GI bleeding, ICU admission and decreased 90-day transplant free survival on multivariate analysis. Our findings suggest that CDC may serve as a clinically significant marker of disease severity in AH and can help guide further clinical trials targeting the gut-liver axis in patients with AH.

## CONFLICTS OF INTEREST

**Guarantor of the article:** Vikrant Rachakonda, MD.

**Specific author contributions:** V.R.: design, conception and supervision of the whole study, data collection, analysis and interpretation, writing initial draft and critical review of final manuscript. J.B.V.: data collection, interpretation, writing initial draft and critical review of final manuscript. I.S.: data collection, critical review of final manuscript. R.L.P.: data collection, interpretation, revision of initial draft and critical review of final manuscript. J.H.: data collection, critical review of final manuscript. S.C.: manuscript. C.R.: data interpretation, critical review of final manuscript.

**Financial support:** None to report.

**Potential competing interests:** None to report.

This study was approved by the Institutional Review Board (IRB) at University of California Davis Medical Center.

**Study Highlights**

**WHAT IS KNOWN**

✓ Alcohol-associated hepatitis is associated with reduced intestinal microbial diversity.
✓ *Clostridioides difficile* is associated with intestinal dysbiosis in chronic diseases.

**WHAT IS NEW HERE**

✓ *C*.* difficile* colonization is associated with increased risk of both *C. difficile* infection and non-*C. difficile* infections in alcohol-associated hepatitis.
✓ *C. difficile *colonization is associated with increased 90-day mortality in alcohol-associated hepatitis, independent of model for end-stage liver diseases (MELD) scores.

## Supplementary Material

## ABBREVIATIONS:

AH: alcohol-associated hepatitis
aHR: adjusted hazards ratio
AKI: acute kidney injury
ALD: alcohol-associated liver disease
ALT: alanine aminotransferase
AST: aspartate aminotransferase
BA: bile acids
CDC: Clostridioides difficile colonization
CDI: Clostridioides difficile infection
DAMP: damage associated molecular pattern
HR: hazards ratio
ICU: intensive care unit
INR: international normalized ratio
LOS: length of stay
MELD: Model for End Stage Liver Diseases
NLR: neutrophil lymphocyte ratio
OR: odds ratio
PCR: polymerase chain reaction
PPI: proton pump inhibitor
PSE: portosystemic encephalopathy
SBP: spontaneous bacterial peritonitis
SSTI: skin/soft tissue infection
UTI: urinary tract infection
WBC: white blood cell
