# Supplementary material for: Impact of Intestinal Colonization With Clostridioides difficile on Clinical Outcomes in Alcohol-Associated Hepatitis
Source: Clin Transl Gastroenterol. 2026 Aug 17;17(10):e01084. doi: 10.14309/ctg.0000000000001084 (PMC13694074; doi:10.14309/ctg.0000000000001084)
Supplement: Supplementary file 2 [file ct9-17-e01084-s001.docx]

***Supplementary Material 1*** *– Definitions of smoking habit, alcohol consumption, and proton pump inhibitor use*

Smoking habit was categorized as: never smoked, ex-smoker, active smoker <5 pack-years, 5–10 pack-years, and >10 pack-years^1^.

Alcohol consumption was classified as abstainers (0 alcohol units/day), light to moderate drinkers (within recommended limits: ≤2 alcohol units/day or ≤14/week for men; ≤1 unit/day or ≤7/week for women), and heavy drinkers (exceeding these thresholds)^2^.

Proton pump inhibitor (PPI) use was grouped into never used, on-demand, short-term or intermittent use (≤ 8 weeks), and chronic use (> 8 weeks)^3^.

1. Liu B, Parsons V, Feuer EJ, et al. Small Area Estimation of Cancer Risk Factors and Screening Behaviors in US Counties by Combining Two Large National Health Surveys. Prev Chronic Dis 2019;16:E119.
2. European Association for the Study of the L. EASL Clinical Practice Guidelines: Management of alcohol-related liver disease. J Hepatol 2018;69:154-181.
3. Targownik LE, Fisher DA, Saini SD. AGA Clinical Practice Update on De-Prescribing of Proton Pump Inhibitors: Expert Review. Gastroenterology 2022;162:1334-1342.

***Supplementary Statistical Methods 1 –*** *Statistical modeling strategy*

Given the relatively small cohort (n=106) and the number of recurrence/progression events (n = 32; 30.2%), particular attention was paid to the events-per-variable (EPV) ratio to minimize the risk of overfitting and unstable estimates. For this reason, no full multivariable model including all candidate predictors was fitted. Instead, multivariable modeling was restricted to parsimonious sensitivity Cox models, and penalized likelihood approaches were used when sparse events or potentially unstable estimates were a concern.

The composite event of recurrence or progression was used to define PFS, which was analyzed as the main time-to-event outcome. Candidate clinical, pathological, and treatment-related predictors were first evaluated using univariate Cox proportional hazards models. Firth’s penalized Cox regression was used as a sensitivity approach for selected models when sparse events or potentially unstable estimates were a concern.

Restricted mean survival time (RMST) up to 60 months was calculated as a complementary summary measure when median survival was not reached, or was poorly interpretable because of the small number of patients remaining at risk in the tail of the Kaplan–Meier curve.

Tumor size was evaluated both as a continuous variable (per 5-mm increase) and as a categorical variable. The categorical cut-off was identified using a time-dependent receiver operating characteristic (ROC) analysis for PFS at 60 months based on the Youden index.

For sensitivity multivariable modeling, tumor size and lesion multiplicity were selected a priori because they represent clinically and biologically relevant dimensions of tumor burden in type 1 gastric neuroendocrine tumors. These parsimonious Cox models were used to assess whether the association between tumor size and PFS remained robust after adjustment for lesion multiplicity. Treatment modality, R0 resection status, sex, tumor grade, and Ki-67 index were not combined into a full multivariable model because the number of events was insufficient to support an adequately powered model including all candidate predictors. Tumor grade and Ki-67 index were not included together in multivariable models because of their conceptual and statistical overlap; moreover, neither variable showed a significant association with PFS in univariate Cox analyses, and adding them to adjusted models would have increased the risk of overfitting relative to the number of events.

In multinomial logistic regression models evaluating factors associated with treatment allocation, statistical significance was assessed using z-tests with robust standard errors.

No outcome data were missing in the dataset.

All statistical analyses were conducted using R (version 4.0.3) with standard packages for survival analysis and penalized regression.

**Supplementary Table 1** – Laboratory Findings at Diagnosis

**Laboratory Parameters**

| **Parameter** | **Median [IQR]** |
| --- | --- |
| Hemoglobin, g/dL [NR: Males 14-18; Females 12-16] | 12.5 [2.7] |
| Mean corpuscular volume, fL  [NR: 80-100] | 86.9 [12.6] |
| Ferritin, µg/L  [NR: Males 40-300; Females 20-200] | 23 [61.0] |
| Vitamin B12, pg/mL  [NR: 200-1000] | 225 [253] |
| Chromogranin A, ng/mL  [NR: 0-100] | 74 [200] |
| Gastrin, pg/mL  [NR: 0-180] | 400 [542] |

**Autoantibodies**

| **Antibody** | **n (%)** |
| --- | --- |
| Anti-parietal cell antibodies | 52 (49%)  NA: 50 (47%) |
| Anti-intrinsic factor antibodies | 10 (9.4%)  NA: 57 (54%) |
| Anti-Helicobacter pylori antibodies | 11 (10.4%)  NA: 52 (49%) |

NR: Normal Range; NA: Not Available; IQR: Interquartile Range
Given the large proportion of patients with missing antibody status, positivity rates should be interpreted with caution, as they may be biased.

**Supplementary Table 2** – Comparison of Clinical and Pathological Characteristics Between Patients With and Without Disease Recurrence/Progression

| **Feature** | **Patients With**  **Recurrence/Progression N (%)** | **Patients Without**  **Recurrence/Progression N (%)** |
| --- | --- | --- |
| **Overall n. of patients** | 32 | 74 |
| **Sex** | F:20 (62.5) / M:12 (37.5) | F:42 (56.7) / M:32 (43.3) |
| **Median [IQR] age at diagnosis (y)** | 57 [16.8] | 59 [22.8] |
| **Median [IQR] tumor size (mm)** | 25 [15.0] | 15 [8.00] |
| **Median [IQR] Ki67** | 2% [2.25] | 2% [4.00] |
| **Grading**  G1  G2  G3 | 22 (68.7)  9 (28.1)  1 (3.2) | 43 (58.1)  31 (41.9)  0 (0) |
| **Number of visible NETs**  Single lesion  2-5 lesions  6-10 lesions  Multiple (number not assessed) | 7 (21.9)  5 (15.6)  8 (25)  12 (37.5) | 26 (35.1)  19 (25.7)  15 (20.3)  14 (18.9) |
| **Treatment**  Snare polypectomy  EMR  m-EMR  ESD  Not specified  Surgery  Surveillance only | 5 (15.6)  11 (34.4)  0 (0)  1 (3.1)  8 (25)  7 (21.9) (4 without previous endoscopic resection)  3 (9.4) | 2 (2.7)  16 (21.6)  6 (8.1)  13 (17.6)  14 (18.9)  26 (35.1)  (16 without previous endoscopic resection)  7 (9.4) |
| **Margin status**  R0  R1  NA | **of 30 pts underwent resection**  13 (43.3)  6 (20)  11 (36.7) | **of 67 pts underwent resection**  49 (73.2)  7 (10.4)  11 (16.4) |

IQR: interquartile range; NA: not available; ESD: Endoscopic Submucosal Dissection; EMR: Endoscopic Mucosal Resection; m-EMR: modified Endoscopic Mucosal Resection

**Supplementary Table 3 –** *Univariate Cox regression analyses for progression-free survival.*

| Analysis set | Predictor | N | Events | HR | 95%CI | p-value |
| --- | --- | --- | --- | --- | --- | --- |
| Overall cohort | Tumor size, per 5-mm increase | 106 | 32 | 1.194 | 1.056–1.351 | .005 |
|  | Tumor size ≥24 mm vs <24 mm | 106 | 32 | 3.928 | 1.868–8.261 | <.001 |
|  | Multiple vs single lesion | 106 | 32 | 1.033 | .440–2.426 | .941 |
|  | Ki-67 index, per 1% increase | 106 | 32 | 1.028 | .944–1.120 | .519 |
|  | Tumor grade, ordinal trend | 106 | 32 | .917 | .442–1.904 | .816 |
|  | Male sex vs female | 106 | 32 | .819 | .400–1.676 | .585 |
|  | Treatment: endoscopic vs surveillance | 106 | 32 | 1.829 | .516–6.481 | .350 |
|  | Treatment: surgical vs surveillance | 106 | 32 | .876 | .222–3.460 | .851 |
| Margin-status subset | R0 vs non-radical resection | 75 | 19 | .383 | .143–1.021 | .055 |
| Endoscopic subgroup | ESD/m-EMR vs other techniques | 46 | 15 | .094 | .012–.713 | .022 |
|  | En-bloc vs non-en-bloc resection | 45 | 14 | .449 | .138–1.466 | .185 |

Hazard ratios (HRs), 95%CIs, and p-values were estimated using univariate Cox proportional hazards models. Progression-free survival was defined as time to recurrence or progression. N indicates the number of patients included in each model, and events indicate the number of recurrence/progression events. Tumor size was modeled both per 5-mm increase and using the ROC-derived 24-mm cutoff. R0, ESD/m-EMR, and en-bloc analyses were restricted to patients with available relevant data.

**Supplementary Figure 1** – *Time-dependent ROC curve for tumor size at 60 months (AUC=0.74)*
