# Supplementary material for: Impact of Intestinal Colonization With Clostridioides difficile on Clinical Outcomes in Alcohol-Associated Hepatitis
Source: Clin Transl Gastroenterol. 2026 Aug 17;17(10):e01084. doi: 10.14309/ctg.0000000000001084 (PMC13694074; doi:10.14309/ctg.0000000000001084)
Supplement: Supplementary file 3 [file ct9-17-e01084-s003.docx]

| **Infections** | **All**  **(N=471)** | **CDC**  **(N=69)** | **NO CDC**  **(N=402)** | p |
| --- | --- | --- | --- | --- |
| **Any infection (n, %)** | 163 (34.6%) | 37 (53.6%) | 126 (31.3%) | 0.001 |
| **CDI (n, %)** | 9 (2.2%) | 6 (8.7%) | 3 (0.7%) | <0.001 |
| **Respiratory Infection (n, %)** | 65 (13.8%) | 14 (21.5%) | 51 (12.7%) | 0.071 |
| **UTI (n, %)** | 49 (10.4%) | 11 (15.9%) | 38 (9.4%) | 0.083 |
| **SBP (n, %)** | 22 (4.7%) | 6 (8.7%) | 16 (4.0%) | 0.086 |
| **NON-CDI ABDOMINAL (n, %)** | 18 (3.8%) | 2 (2.9%) | 16 (4.0%) | 0.494 |
| **SSTI (n, %)** | 14 (3.0%) | 0 (0.0%) | 14 (3.5% | 0.105 |
| **Bacteremia, (n, %)** | 29 (6.2%) | 5 (7.2%) | 24 (6.0%) | 0.424 |

**Supplemental Table 1:** Rate of infections stratified by *C. difficile* colonization status. CDC, *C. difficile* colonization; CDI, *C. difficile* infection; SBP, spontaneous bacterial peritonitis; SSTI, skin or soft tissue infection; UTI, urinary tract infection

| **Feature (90d mortality, severe AH only)** | **UV HR** | **p** | **MV HR** | p |
| --- | --- | --- | --- | --- |
| **Age** | 1.026 (1.007 – 1.044) | 0.006 | 1.029 (1.010 – 1.049) | 0.002 |
| **Sex** | 0.880 (0.579 – 1.338) | 0.551 |  |  |
| **White** | 1.105 (0.734 – 1.664) | 0.633 |  |  |
| **ICU** | 2.286 (1.500 – 3484) | <0.001 | 1.589 (1.027 – 2.460) | 0.037 |
| **Platelets** | 0.996 (0.997 – 1.002) | 0.753 |  |  |
| **Ascites** | 2.063 (1.168 – 3.645) | 0.013 | 1.390 (0.746 – 2.589) | 0.300 |
| **Hepatic Encephalopathy** | 1.712 (1.131 – 2.594) | 0.011 | 1.175 (0.753 – 1.830) | 0.478 |
| **Variceal Bleed** | 2.023 (1.343 – 3.047) | 0.001 | 1.385 (0.881 – 2.175) | 0.158 |
| **AKI** | 1.921 (1.274 – 2.896) | 0.002 | 1.023 (0.651 – 1.607) | 0.922 |
| **MELD 3.0** | 1.090 (1.065 – 1.115) | <0.001 | 1.086 (1.060 – 1.114) | <0.001 |
| **Abx** | 2.095 (1.014 – 4.327) | 0.046 | 0.899 (0.402 – 2.012) | 0.796 |
| **Infection** | 1.961 (1.298 – 2.962) | 0.001 | 1.311 (0.828 – 2.074) | 0.248 |
| **WBC** | 1.012 (0.989 – 1.033) | 0.324 |  |  |
| **NLR** | 1.010 (0.995 – 1.025) | 0.205 |  |  |
| **CDC** | 2.111 (1.316 – 3.387) | 0.002 | 1.866 (1.153 – 3.005) | 0.010 |

**Supplemental Table 2:** Univariate and multivariate Cox proportional hazards models for factors associated with 90-day survival among patients with successfully completed toxigenic *C. difficile* colonization testing and severe AH, defined as MELD > 20 (n=285). AKI, acute kidney injury CDC, *C. difficile* colonization; ICU, intensive care unit; LOS, length of stay; NLR, neutrophil-lymphocyte raito; PPI, proton pump inhibitor; WBC, white blood cell count.
